# Clinical characteristics and factors associated with severe acute respiratory infection and influenza among children in Jingzhou, China

**DOI:** 10.1111/irv.12419

**Published:** 2016-09-20

**Authors:** Yang Huai, Xuhua Guan, Shali Liu, Timothy M. Uyeki, Hui Jiang, John Klena, Jigui Huang, Maoyi Chen, Youxing Peng, Hui Yang, Jun Luo, Jiandong Zheng, Zhibin Peng, Xixiang Huo, Lin Xiao, Hui Chen, Yuzhi Zhang, Xuesen Xing, Luzhao Feng, Dale J. Hu, Hongjie Yu, Faxian Zhan, Jay K. Varma

**Affiliations:** ^1^ China‐US Collaborative Program on Emerging and Re‐Emerging Infection DiseaseCenter for Global HealthCenters for Disease Control and PreventionBeijingChina; ^2^ Hubei Provincial Centre for Disease Control and PreventionWuhanChina; ^3^ Jingzhou Central HospitalJingzhouChina; ^4^ Influenza DivisionNational Center for Immunization and Respiratory DiseasesAtlantaGAUSA; ^5^ Key Laboratory of Surveillance and Early‐warning on Infectious DiseaseDivision of Infectious DiseaseChinese Center for Disease Control and PreventionBeijingChina; ^6^ Global Disease Detection BranchDivision of Global Health ProtectionCenter for Global HealthCenters for Disease Control and PreventionAtlantaGAUSA; ^7^ Jingzhou Center for Disease Control and PreventionJingzhouChina; ^8^ Jingzhou First People's HospitalJingzhouChina; ^9^ Jingzhou Second People's HospitalJingzhouChina; ^10^ Jingzhou Maternal and Children's HospitalJingzhouChina; ^11^ Vaccine Clinical Research Branch Vaccine Research ProgramDivision of AIDSNIAID/NIHBethesdaMDUSA

**Keywords:** children, influenza, risk factors, severe acute respiratory infection

## Abstract

**Background:**

Influenza is an important cause of respiratory illness in children, but data are limited on hospitalized children with laboratory‐confirmed influenza in China.

**Methods:**

We conducted active surveillance for severe acute respiratory infection (SARI; fever and at least one sign or symptom of acute respiratory illness) among hospitalized pediatric patients in Jingzhou, Hubei Province, from April 2010 to April 2012. Data were collected from enrolled SARI patients on demographics, underlying health conditions, clinical course of illness, and outcomes. Nasal swabs were collected and tested for influenza viruses by reverse transcription polymerase chain reaction. We described the clinical and epidemiological characteristics of children with influenza and analyzed the association between potential risk factors and SARI patients with influenza.

**Results:**

During the study period, 15 354 children aged <15 years with signs and symptoms of SARI were enrolled at hospital admission. severe acute respiratory infection patients aged 5–15 years with confirmed influenza (H3N2) infection were more likely than children without influenza to have radiographic diagnosis of pneumonia (11/31, 36% vs 15/105, 14%. *P*<.05). Only 16% (1116/7145) of enrolled patients had received seasonal trivalent influenza vaccination within 12 months of hospital admission. Non‐vaccinated influenza cases were more likely than vaccinated influenza cases to have pneumonia (31/133, 23% vs 37/256, 15%, *P*<.05). severe acute respiratory infection cases aged 5–15 years diagnosed with influenza were also more likely to have a household member who smoked cigarettes compared with SARI cases without a smoking household member (54/208, 26% vs 158/960, 16%, *P*<.05).

**Conclusions:**

Influenza A (H3N2) virus infection was an important contributor to pneumonia requiring hospitalization. Our results highlight the importance of surveillance in identifying factors for influenza hospitalization, monitoring adherence to influenza prevention and treatment strategies, and evaluating the disease burden among hospitalized pediatric SARI patients. Influenza vaccination promotion should target children.

## 1 Background

Influenza is an important vaccine‐preventable infectious disease that causes significant morbidity worldwide in persons of all ages. Studies conducted in North America, Europe, and Asia have documented that children have particularly high rates of morbidity and influenza‐related complications, particularly those aged <2 years.^1^, ^2^, ^3^ Although effectiveness varies by season through the interaction of viral and host factors, annual influenza vaccination remains the most effective method for preventing seasonal influenza and related complications.^4^, ^5^, ^6^, ^7^ In the United States, annual influenza vaccination is recommended for all persons aged ≥6 months of age.^8^ In China, availability and use of seasonal influenza vaccine has increased dramatically; nevertheless, the vast majority of Chinese children are not vaccinated annually.^9^

During 2010, in the post‐2009 H1N1 pandemic period, we initiated active surveillance for severe acute respiratory infection (SARI) and influenza in a central Chinese city. The highest influenza‐associated SARI rates occurred among children aged 6–11 months (3603 and 3805 hospitalizations per 100 000 during 2010–2011 and 2011–2012, respectively) and influenza‐associated SARI mostly affected children aged <5 years (2021 hospitalizations per 100 000 during 2010–2011 and 2349 per 100 000 during 2011–2012).^10^ We conducted active SARI surveillance in central China to analyze the clinical and epidemiological characteristic of influenza‐associated SARI cases, including pneumonia, among pediatric patients aged <15 years.

## 2 Methods

Surveillance was conducted in Jingzhou, Hubei Province, in three general hospitals and one pediatric hospital as previously described.^10^ All patients admitted to a surveillance hospital were screened by nurses and physicians for SARI and were considered eligible if the SARI case definition was met within 24 hours of hospital admission. Briefly, we collected demographic, clinical, and outcome data and performed influenza testing for all pediatric patients (aged <15 years) hospitalized for SARI to characterize the epidemiology of severe influenza in children.

### 2.1 Case definitions

Children <15 years of age met the case definition for SARI if they had measured elevated temperature (rectal or axillary) ≥37.3°C and at least one sign or symptom of acute respiratory illness, including cough, sore throat, tachypnea, difficulty breathing, abnormal breath sounds on auscultation, sputum production, hemoptysis, chest pain, or chest radiograph consistent with pneumonia. A laboratory‐confirmed influenza case was defined as a SARI patient with a documented positive result of real‐time reverse transcription polymerase chain reaction (RT‐PCR) assay for influenza, admitted to a participating hospital between April 5, 2010, and April 8, 2012. A pneumonia case was defined as a SARI patient with infiltrates noted on chest radiograph performed at any time during the hospitalization.

### 2.2 Data collection

Physicians screened potential case patients for SARI at hospital admission and obtained verbal consent from a parent or guardian for enrollment in the study. Physicians then abstracted data from the medical records of patients meeting the SARI case definition including basic demographic information, past medical history, signs, symptoms, and radiographic results on a structured case report form. At hospital discharge, physicians were required to update the form to include data about outcome and influenza testing results.

### 2.3 Laboratory

Nurses collected nasal swabs from pediatric SARI case patients within 24 hours of admission following standardized procedures and were transferred to the Jingzhou Center for Disease Control and Prevention (Jingzhou CDC). At Jingzhou CDC, laboratory confirmation of influenza virus infection was determined by real‐time RT‐PCR. Handling and testing of specimens has been described in detail elsewhere.^10^

### 2.4 Analysis

Data were entered into epidata (version 2.0, Odense, Denmark). Descriptive statistics including frequency analysis for categorical variables, medians, and interquartile ranges (IQRs) for continuous variables were calculated. To further examine the association between potential risk factors and SARI, we first performed univariate analysis. For multivariable logistic regression, we included variables with *P*<.10 in univariate analysis or those believed to be potential risk factors associated with the outcome of interest, such as “at least one tobacco smoker in household” and we conducted stepwise backwards variable selection, retaining variables with *P*<.05 after controlling for other risk factors in the logistic regression model. Data were analyzed with spss (v17.0; SPSS, Chicago, IL, USA).

### 2.5 Human subjects review

This project was approved by the ethical review committees at the Chinese Center for Disease Control and Prevention (China CDC, Beijing, China) and the Centers for Disease Control and Prevention (US CDC, Atlanta, GA, USA). In response to the 2009 H1N1 pandemic, China's Ministry of Health implemented the 2nd edition of the national protocol in October 2009 to conduct surveillance for influenza‐associated hospitalizations and authorized participating hospitals to collect individual patient data and specimens from patients hospitalized with acute respiratory infection. For this project, therefore, participation only required patients or their parent/guardian to provide brief verbal consent.

## 3 Results

### 3.1 Overall

**Figure 1:**
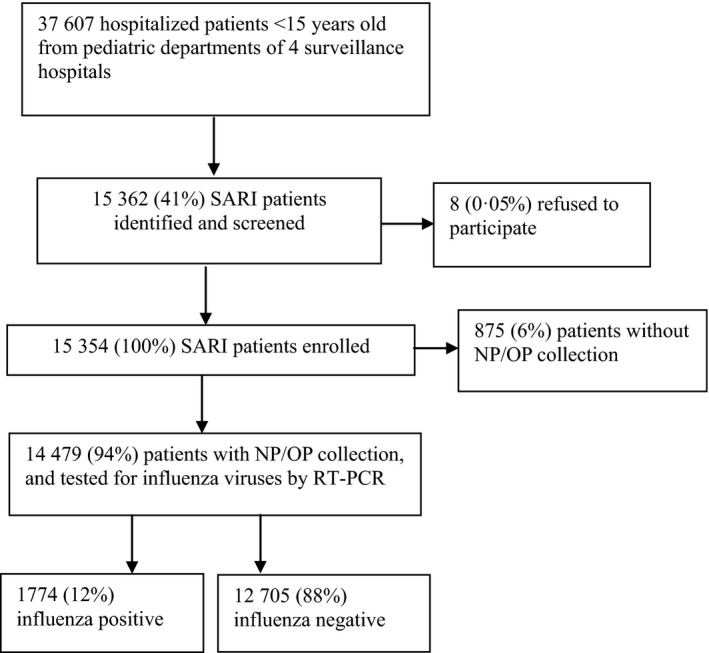
Flowchart of case enrollment in active surveillance for severe acute respiratory infection among hospitalized pediatric patients in Jingzhou

From April 5, 2010, to April 8, 2012, a total of 37 607 patients aged <15 years were hospitalized in four surveillance hospitals in Jingzhou, and 15 354 (41%) of them met the SARI case definition and 15 354 were enrolled (0.05% refused; Fig. 1). Over the 24‐month period, there were increases in influenza activity during summer (August and September), winter (December–February), and spring (March–May) months.

### 3.2 SARI patients

The median age of all enrolled SARI patients was 1.9 years (interquartile range [IQR], 0.9–3.3 years), 80% were aged <5 years, and 57% were male. Only 2% of SARI patients had an underlying medical condition, the most common of which was prematurity (2%). Only 1% of influenza patient received monovalent pandemic H1N1 vaccination and 16% patient received seasonal trivalent influenza vaccination (Table 1). severe acute respiratory infection patients were admitted to the hospital a median of 2 days (IQR, 1–3 days) after illness onset. Cough (61%) and sore throat (27%) were the most frequent symptoms of SARI patients at hospital admission. A quarter of all SARI patients had high fever (temperature ≥39.0**°**C) at admission. The median length of hospitalization for SARI patients was 5 days (IQR, 3–6). Of these SARI patients, 3 (0.02%) died during hospitalization, and 5 (0.03%) died within 30 days after discharge (Table 2).

**Table 1:** Demographic characteristics and underlying medical conditions of pediatric patients with severe acute respiratory infection with and without laboratory‐confirmed influenza, Jingzhou, China, April 5, 2010 to April 8, 2012

Characteristics^a^	Confirmed Influenza (n=1774) (%)	Influenza negative (n=12 705) (%)	*P*‐value^b^	Total^b^ (n=15 354) (%)
Male sex	1024 (58)	7248 (57)	.591	8789 (57)
Median (IQR) age (years)	2.5 (1.0–4.3)	1.8 (0.9–3.2)	**<.05**	1.9 (0.9–3.3)
Age group:				
<6 mo	156 (9)	1099 (9)		1318 (9)
6–11 mo	263 (15)	2684 (21)		3121 (20)
12–23 mo	339 (19)	3042 (24)		3596 (23)
2–4 y	669 (38)	4361 (34)		5318 (35)
5–9 y	300 (17)	1300 (10)		1710 (11)
10–15 y	47 (3)	219 (2)		291 (2)
At least one underlying medical condition	39 (2)	283 (2)	.938	333 (2)
Asthma	3 (0.2)	34 (0.3)	.617	39 (0.3)
Chronic bronchitis	0	8 (0.1)	.607	9 (0.1)
Obesity^c^	4/1016 (0.4)	16/5880 (0.3)	.833	22/7319 (0.3)
Prematurity^d^	17/758 (2)	97/6825 (1)	.507	117/8035 (2)
Low birth‐weight^e^	13/758 (2)	67/6825 (1)	.862	82/8035 (1)
Received Influenza vaccination^f^				
Received monovalent pandemic H1N1 vaccination^g^	5/407 (1)	76/2299 (3)	.778	83/2826 (3)
Received seasonal trivalent influenza vaccination^h^	151/959 (16)	1116/7145 (16)	.907	1311/8528 (15)
Exposure history				
At least one tobacco smoker in household	308/1182 (26)	1865/8928 (21)	**<.05**	2294/10 640 (22)
Sick contact with fever or respiratory symptoms^i^	82/1083 (8)	390/8078 (5)	**<.05**	497/9601 (5)
More than one child living in household in past 3 mo	60 (3)	344 (3)	.106	422 (3)

IQR, interquartile range.

a Data presented as no. (%) of patients unless otherwise indicated. Denominators for testing of fewer patients than full group are indicated. Percentages may not total 100 because of rounding. Data did not include patients without specimens for influenza virus testing.

The *P*‐values are comparisons between “pediatric SARI patients with confirmed influenza infection” and “pediatric SARI patients without influenza infection”, and statistically significant as *P*<.05

b There were 875 (6%) patients without NP/OP collection.

c Body mass index (BMI) was calculated for patients with available height and weight data to assess obesity using Chinese criteria (BMI greater than the cutoff values for children aged 2–17 y). BMI was not calculated in children aged <2 y.^11^

d Defined as gestational age <37 wk at birth for children aged <2 y.

e Defined as birth weight of a live born infant of <2500 g for children aged <2 y.

f Answered “Yes” to this question, “During the past 12 mo, have you had a flu shot?” which were asked during a face‐to‐face interview.

g Of children aged ≥6 mo eligible to receive monovalent pandemic A (H1N1) influenza vaccine.

h Of children aged ≥6 mo eligible to receive seasonal trivalent inactive influenza vaccine.

i Contact with anyone else with fever or respiratory symptoms defined as having been in close contact (within 1 m) or direct contact of person with fever or respiratory symptoms in the previous 2 wk.

**Table 2:** Clinical characteristics of pediatric patients with severe, acute respiratory infection with and without laboratory‐confirmed influenza, Jingzhou, China, April 5, 2010 to April 8, 2012

Characteristics^a^	Confirmed Influenza (n=1774) (%)	Negative for Influenza (n=12 705) (%)	*P*‐value b	Total (n=15 354)^c^ (%)
Symptoms at hospital admission				
Cough	1157 (65)	7205 (57)	**<.05**	8836 (61)
Sore throat	401 (23)	3531 (28)	**<.05**	4098 (27)
Rhinorrhoea	323 (18)	1748 (14)	<**.05**	2192 (14)
Sputum production	135 (8)	1089 (9)	.173	1304 (9)
Dyspnoea	39 (2)	556 (4)	**<.05**	642 (4)
Vomiting	69 (4)	721 (6)	**<.05**	859 (6)
Diarrhea	28 (2)	540 (4)	**<.05**	609 (4)
Abdominal pain	27 (2)	186 (2)	.849	228 (2)
Signs at hospital admission				
Temperature (*T*, °C)				
*T*<36.0	0	6 (0.05)	**<.05**	7 (0.05)
36.0≤*T*<37.0	251 (14)	2071 (6)		2460 (16)
37.0≤*T*<38.0	373 (21)	3048 (24)		3624 (24)
38.0≤*T*<39.0	676 (38)	4461 (35)		5459 (36)
*T*≥39.0	474 (27)	3119 (25)		3804 (25)
Abnormal breath sounds on auscultation	537 (30)	3799 (30)	.751	4579 (30)
Tachypnea	1 (0.1)	25 (0.2)	.361	29 (0.2)
Pneumonia Diagnosis				
Clinically diagnosed as pneumonia at hospital admission	238 (13)	1911 (15)	.071	2291 (15)
Clinically diagnosed as pneumonia at hospital admission and chest radiograph performed	195/238 (82)	1623/1911 (85)	.227	1938/2291 (85)
Radiographic diagnosis of pneumonia	161/195 (83)	1401/1623 (86)	.154	1674/1938 (86)
Chest radiograph performed at hospital admission and during hospitalization	807 (46)	5600 (44)	.262	6841 (45)
Radiographic diagnosis of pneumonia	246/807 (30)	2188/5600 (39)	**<.05**	2583/6841 (38)
Clinical laboratory findings				
Leucopenia^d^	165/1525 (11)	557/11 055 (5)	**<.05**	777/13 299 (6)
Leukocytosis^e^	282/1525 (19)	3657/11 055 (33)	**<.05**	4188/13 299 (31)
Anemia^f^	269/1520 (18)	2313/11 014 (21)	**<.05**	2748/13 250 (21)
Elevated erythrocyte Sedimentation rate^g^	66/158 (42)	641/1086 (59)	**<.05**	777/1349 (58)
C‐reactive protein >8 mg/L	117/476 (25)	1431/3397 (42)	**<.05**	1622/4040 (40)
Treatment				
Received antibiotics before hospital admission	670/1343 (50)	4529/9831 (46)	**<.05**	5490/11 802 (47)
Received antibiotics during hospitalization	1750 (99)	12 461 (98)	.097	15 056 (98)
Received corticosteroids during hospitalization	164 (9)	1591 (13)	**<.05**	1880 (12)
Received oseltamivir during hospitalization	2 (0.1)	21 (0.2)	1.000	23 (0.1)
Supplemental oxygen required	16 (0.9)	183 (1.4)	.086	216 (1)
Mechanical ventilation	1 (0.1)	2 (0.05)	.324	5 (0.1)
Admitted to ICU	1 (0.1)	18 (0.1)	.722	21 (0.1)
Complications				
Neurologic disorders	3 (0.2)	11 (0.1)	.241	20 (0.1)
Respiratory failure	1 (0.1)	8 (0.1)	1.000	10 (0.1)
Cardiac failure	1 (0.1)	11 (0.1)	1.000	14 (0.1)
Hepatic dysfunction	2 (0.1)	3 (0.01)	.117	7 (0.1)
Clinical course, median days (IQR)				
From illness onset to hospital admission	2 (1–3)	2 (1–3)	.860	2 (1–3)
From hospital admission to discharge or death	5 (3–6)	5 (3–6)	.936	5 (3–6)
Clinical outcome				
Died during hospitalization	0	3 (0.02)	.221	3 (0.02)
Died within 30 d after discharge	1 (0.1)	4 (0.03)	.480	5 (0.03)

ICU, intensive care unit; IQR, interquartile range.

a Data presented as no. (%) of patients unless otherwise indicated. Denominators for testing of fewer cases than full group are indicated. Percentages may not total 100 because of rounding. Data only include patients who provided specimens for influenza virus testing.

b The *P*‐values are comparisons between “pediatric SARI patients with confirmed influenza infection” and “pediatric SARI patients without influenza infection”, and statistically significant as *P*<.05.

c There were 875 (6%) patients without NP/OP collection.

d Defined as a reduction in the circulating WBC count to <4000/μL.

e Defined as a reduction in the circulating WBC count to >10 000/μL.

f Defined as a reduction in the hemoglobin in grams per liter (Hb<110 g/L).

g Raised erythrocyte sedimentation rate defined as >20 mm/h.

### 3.3 SARI patients with laboratory‐confirmed influenza

Of 14 479 (14 479/15 354, 94%) children who met the SARI case definition and had respiratory specimens collected, 1774 (17, 74/14, 479, 12%) tested positive for influenza viruses. Of the 1774 children with confirmed influenza, 58% were male and 88% were aged <5 years. Nearly all influenza‐associated SARI cases were previously healthy, and only 2% had at least one underlying medical condition, the most common of which were low birth weight (1%) and prematurity (1%) among children aged <2 years.

Influenza‐associated SARI cases were admitted to the hospital a median of 2 days (IQR, 1–3 days) after illness onset, and presented with fever (temperature ≥37.3°C; 82%), cough (65%), and sore throat (23%; Table 2). Other symptoms included rhinorrhea (18%) and sputum production (8%). Gastrointestinal symptoms (nausea, vomiting, and diarrhea) were uncommon. Twenty‐seven percent of influenza cases had high fever (temperature ≥39.0°C) at admission. Complications among influenza‐associated SARI patients were very uncommon, but included neurologic disorders (0.2%), respiratory failure (0.1%), cardiac failure (0.1%), and hepatic dysfunction (0.1%). Twenty‐one influenza patients were admitted to an intensive care unit, and five required mechanical ventilation. The median length of hospitalization for influenza‐associated SARI patients was 5 days (IQR, 3–6); of these, 1 (0.06%) died within 30 days after discharge.

### 3.4 SARI patients with and without laboratory‐confirmed influenza

Of 14 479 children who met the SARI case definition and had respiratory specimens collected during the study period, 12 705 (88%) tested negative for influenza viruses. The demographic characteristics of patients with and without influenza were similar except those without influenza had a lower median age and a younger age distribution (*P*<.05; Table 1). Influenza cases were more likely to have at least one tobacco smoker in the household and a sick contact with fever or respiratory symptoms than children without influenza (*P*<.05).

Clinical characteristics of pediatric influenza cases and children without influenza were similar. More pediatric influenza cases had cough and rhinorrhea than children without influenza (*P*<.05), while more SARI cases without influenza had gastrointestinal symptoms (nausea, vomiting, and diarrhea) than influenza cases (Table 2). More influenza cases had high fever at hospital admission than those without influenza (*P*<.05).

### 3.5 SARI cases with pneumonia

Of 2291 SARI patients clinically diagnosed with pneumonia at hospital admission, 1938 (85%) had chest radiography performed, and 1674 (86%) had evidence of pneumonia. The median length of hospitalization for pneumonia cases was 6 days (IQR, 5–8), which was longer (*P*<.05) than children without pneumonia (median 5 days, IQR, 3–6). Among influenza cases, the proportion with cough, sputum production, or Dyspnoea was higher in children with radiologic evidence of pneumonia compared with those without (*P*<.05; Table 3). However, children without radiologic evidence of pneumonia had higher temperatures (*T*>38°C) on admission than children with radiologic evidence of pneumonia, but more pneumonia cases had abnormal breath sounds on auscultation than children without pneumonia (*P*<.05).

**Table 3:** Clinical characteristics of pediatric severe, acute respiratory infection patients with laboratory‐confirmed influenza with and without radiographic diagnosis of pneumonia in Jingzhou, China, April 5, 2010 to April 8, 2012

Characteristics of influenza patients^a^	Case patients with pneumonia (n=246) (%)	Case patients without pneumonia (n=561) (%)	*P*‐value b
Symptoms at hospital admission			
Cough	209 (85)	341 (61)	**<.05**
Sore throat	48 (20)	111 (20)	.928
Rhinorrhoea	40 (16)	103 (18)	.472
Sputum	43 (18)	56 (10)	**<.05**
Dyspnoea	19 (8)	11 (2)	**<.05**
Vomiting	4 (2)	25 (5)	.062
Diarrhea	0	11 (2)	.022
Abdominal pain	0	8 (1)	.115
Signs at hospital admission			
Temperature (*T*, °C)			
*T*<36.0	0	0	**<.05**
36.0≤*T*<37.0	51 (21)	100 (18)	
37.0≤*T*<38.0	62 (25)	124 (22)	
38.0≤*T*<39.0	82 (33)	202 (36)	
*T*≥39.0	51 (21)	135 (24)	
Abnormal breath sounds on auscultation	154 (63)	191 (34)	**<.05**
Tachypnea	1 (0.4)	0	.305
Clinical laboratory findings			
Leucopenia^c^	14/228 (6)	60/493 (12)	**<.05**
Leukocytosis^d^	42/228 (18)	85/493 (17)	.753
Anemia^e^	50/229 (22)	80/492 (16)	.070
Raised erythrocyte sedimentation rate^f^	16/33 (49)	28/72 (39)	.355
C‐reactive protein>8 mg/L	24/94 (26)	34/154 (22)	.533
Influenza virus type/subtype			
Influenza B	101 (41)	237 (42)	.753
A(H1N1)pdm09	46 (19)	88 (16)	.290
A (H3N2)	99 (40)	236 (42)	.642
Treatment			
Received antibiotics before hospital admission	112/177 (63)	217/411 (53)	**<.05**
Received antibiotics during hospitalization	245 (100)	558 (100)	.811
Received corticosteroids during hospitalization	35 (14)	54 (10)	.055
Received oseltamivir during hospitalization	1 (0.4)	1 (0.2)	.517
Supplemental oxygen required	4 (2)	6 (1)	.503
Mechanical ventilation	1 (0.4)	0	.131
Complications			
Neurologic disorders	1 (0.4)	1 (0.2)	.517
Respiratory failure	1 (0.4)	0	.305
Cardiac failure	1 (0.4)	0	.305
Hepatic dysfunction	1 (0.4)	0	.305
Clinical course, median days (IQR)			
From illness onset to hospital admission	3 (1–5)	2 (0–3)	**<.05**
From hospital admission to discharge or death	6 (5–8)	5 (3–6)	**<.05**
Died during hospitalization	0	0	−
Died within 30 d after discharge	0	1 (0.2)	1.000

ICU, intensive care unit; IQR, interquartile range.

a Data presented as no. (%) of patients unless otherwise indicated. Denominators for testing of fewer cases than full group are indicated. Percentages may not total 100 because of rounding. Data only include patients with specimens for influenza virus testing.

b The *P*‐values are comparisons between “pediatric influenza patients with pneumonia” and “pediatric influenza patients without pneumonia”, and statistically significant as *P*<.05.

c Defined as a reduction in the circulating WBC count to <4000/μL.

d Defined as a reduction in the circulating WBC count to >10 000/μL.

e Defined as a reduction in the hemoglobin in grams per liter (Hb<110 g/L).

f Raised erythrocyte sedimentation rate defined as exceed up limit as 20 mm/h.

Of 807 SARI cases with radiologic evidence of pneumonia that were tested for influenza viruses, 246 (30%) were positive, including 145 (59%) with influenza A and 101 (41%) with influenza B (Table 4). Among the influenza A cases, 46 (32%) were due to A(H1N1)pdm09 and 99 (68%) were due to A(H3N2). Among pneumonia cases aged 5–15 years, the proportion associated with A(H3N2) was significantly higher than either A(H1N1)pdm09 or influenza B (Table S3‐1).

**Table 4:** Demographic characteristics and underlying medical conditions of pediatric severe, acute respiratory infection patients with laboratory‐confirmed influenza with and without radiographic diagnosis of pneumonia, Jingzhou, China, April 5, 2010 to April 8, 2012

Characteristics of influenza patients^a^	Case patients with pneumonia (n=246) (%)	Case patients without pneumonia (n=561) (%)	*P*‐value^b^
Male sex	145 (59)	332 (59)	.950
Median (IQR) age (years)	2.3 (0.9–3.7)	2.2 (1.0–4.0)	.542
Age group:			
<6 mo	29 (12)	50 (9)	
6–11 mo	38 (15)	87 (16)	
12–23 mo	43 (18)	124 (22)	
2–4 y	105 (43)	195 (35)	
5–9 y	26 (11)	90 (16)	
10–15 y	5 (2)	15 (3)	
At least one underlying medical condition	7 (3)	9 (2)	.275
Prematurity^c^	2/110 (2)	4/261 (2)	.744
Low birthweight^d^	1/110 (1)	5/261 (2)	.705
Vaccination history^e^			
Received monovalent pandemic H1N1 vaccination^f^	0	2/132 (2)	1.000
Received seasonal trivalent influenza vaccination^g^	31/133 (23)	37/256 (15)	**<.05**
Exposure history			
At least one tobacco smoker in household	37/160 (23)	102/322 (32)	.051

IQR, interquartile range.

a Data presented as no. (%) of patients unless otherwise indicated. Denominators for testing of fewer patients than full group are indicated. Percentages may not total 100 because of rounding. Data did not include patients without specimens for influenza virus testing.

b The *P*‐values are comparisons between “pediatric influenza patients with pneumonia” and “pediatric influenza patients without pneumonia”, and statistically significant as *P*<.05.

c Defined as gestational age <37 wk at birth for children aged <2 y.

d Defined as birth weight of a live born infant of <2500 g for children aged <2 y.

e Answered “Yes” to this question, “During the past 12 mo, have you had a flu shot?” which were asked during a face‐to‐face interview.

f Of children aged ≥6 mo eligible to receive seasonal trivalent inactive influenza vaccine.

g Of children aged ≥6 mo eligible to receive monovalent pandemic A (H1N1) influenza vaccine.

### 3.6 Treatment

Forty‐seven percent of SARI patients had either been prescribed an antibiotic or self‐administered an antibiotic before hospitalization. A higher proportion of influenza‐associated SARI cases had been prescribed an antibiotic before admission than those without influenza (Table 2, *P*<.05). Nearly all SARI patients were treated with antibiotics after hospital admission regardless of influenza testing results. Fewer cases of influenza‐associated SARI were prescribed corticosteroids during hospitalization than those without influenza (*P*<.05) and 35 (21%) of them had developed X‐ray confirmed pneumonia (*P*<.05), which was significantly higher than those influenza cases without cortisosteroid treatment. Only 0.1% of SARI cases with laboratory‐confirmed influenza received oseltamivir treatment during hospitalization.

### 3.7 Risk factors associated with influenza

The results of univariate and multivariable analyses of potential factors associated with influenza among SARI patients are presented in Tables S1‐1–S8. The proportion of SARI cases with a household member who smokes cigarettes in the home or contact with anyone with fever or respiratory symptoms was significantly associated with influenza among young children aged 6–23 months. These two factors were also significantly associated with higher odds of influenza for children aged 2–4 years and children aged 5–15 years. In multivariable analyses, low birthweight (OR, 6.081; 95% CI, 1.308–28.266) and living with someone who smokes cigarettes in the household (OR, 1.363; 95% CI, 1.047–1.776) were independent risk factors for increased odds of influenza among children with SARI (Table S8).

## 4 Discussion

To our knowledge, this is one of the first prospective studies describing laboratory‐confirmed influenza among hospitalized children in China. We used data from an active, population‐based surveillance system to assess epidemiological and clinical characteristics of hospitalized children with influenza. Our results are consistent with our recent disease burden estimation, which found that influenza was associated with an estimated 2021 SARI hospitalizations per 100 000 during 2010–2011 and 2349 per 100 000 during 2011–2012 among children aged <5 years^10^ and other studies outside China.^12^, ^13^ In this study, we observed the low birth weight and living with someone who smokes cigarettes in the household were independent risk factors for increased odds of influenza among children with SARI. We also documented a low percentage (1%–16%) of SARI patients with influenza who had received influenza vaccine. Low antiviral treatment of influenza and high inappropriate use of antibiotics and corticosteroids were identified from the study.

In 2012, the WHO Strategic Advisory Group of Experts on immunization suggested that children up to 5 years of age should be considered as a target group for annual influenza vaccination.^14^ Several national guidelines recommend annual influenza immunization for children.^15^, ^16^, ^17^ However, the vast majority of the Chinese population is not vaccinated annually. Several studies have estimated vaccine effectiveness across influenza seasons and patient care settings using laboratory‐confirmed influenza cases and found a significant protective effect for children.^18^, ^19^, ^20^, ^21^, ^22^, ^23^ In our study, we did not document types or doses of vaccine administered, making it difficult to draw conclusions about vaccine effectiveness.

Almost no children with influenza were treated with oseltamivir, even though the drug is well tolerated in children and treatment started within 24 hours of symptom onset provides substantial benefits to children with influenza A infection.^24^ It is urgent to make oseltamivir more widely available for influenza patients, and educate Chinese clinicians about the clinical benefit of early oseltamivir treatment. More widespread use of rapid molecular assays with high sensitivity could also increase the proportion of children appropriately treated for influenza.

Corticosteroids were prescribed to 9% of influenza patients in this study. In a retrospective cohort study of hospitalized patients with A (H1N1)pdm09 influenza virus infection in China, the early use of glucocorticoids for fever reduction and pneumonia prevention was associated with the subsequent development of critical illnesses, even after adjusting for the presence of underlying diseases or risk factors.^25^, ^26^ Corticosteroids may also prolong influenza viral shedding in the respiratory tract.^27^ Based on available data, WHO does not currently recommend the use of systemic high‐dose corticosteroids for influenza‐associated pneumonia.^28^

While the infections (12%) in this study were caused by influenza viruses, clinicians prescribed antibiotics for almost all SARI patients. While we did not have data to assess the need for antibiotics in our study population, inappropriate antibiotic use is potentially harmful to the community, fostering the growth of antimicrobial‐resistant organisms.^29^ Reducing inappropriate antibiotic use among children in China may be challenging. Physicians may not be willing to wait for viral testing results, influenza testing is not widely available, and, even when widely available as in this study, physicians often did not receive results until at least 48 hours after specimen collection. Parents may also be less willing to accept waiting for test results before antibiotic administration. Indeed, over half of SARI patients were already treated with antibiotics before hospital admission.^30^

Severe acute respiratory infection patients exposed to tobacco smoke were more likely to have flu as an etiology of SARI. Environmental tobacco smoke or secondhand smoke causes ill health and mortality in children, especially among those under 5 years of age.^31^, ^32^, ^33^ Exposure to secondhand smoke kills approximately 100 000 people every year in China.^34^ Reducing secondhand smoke exposure is one of four priorities identified by the World Health Organization for global tobacco prevention and control. China's 12th 5‐Year Plan calls for smoke‐free public places as part of the major national goal to increase life expectancy. Despite the significant progress made in tobacco control in China, many children are still exposed to secondhand smoke in their home.

This study is subject to several limitations. First, our study only captured children who met our surveillance case definition for severe acute respiratory infections. Young infants might have fever only without any respiratory symptoms. Second, our study was only able to test for influenza virus infection, and only 12% of pediatric SARI patients had evidence of influenza virus infection. We do not know the etiology of illness among most pediatric SARI patients enrolled in our study.

## 5 Conclusions

Our results highlight the importance of surveillance in identifying factors associated with influenza in hospitalized SARI patients, monitoring adherence to influenza prevention and treatment strategies, and evaluating the year‐round disease burden during influenza seasons in central China. Additional research on pediatric SARI patients in China should also incorporate testing for other respiratory pathogens, further elucidate the connection between tobacco exposure and outcomes of SARI, and identify factors that can help clinicians rapidly start antiviral treatment of influenza while reducing inappropriate use of antibiotics and corticosteroids.

## Financial Support

This study was supported by the Cooperative Agreement Number, 5U2GGH000018, funded by the Centers for Disease Control and Prevention. Its contents are solely the responsibility of the authors and do not necessarily represent the official views of the Centers for Disease Control and Prevention or the Department of Health and Human Services.

## Conflicts of Interest

We declare that we have no conflict of interests.

## References

[1] Thompson WW , Shay DK , Weintraub E , et al. Influenza‐associated hospitalizations in the United States. JAMA. 2004;292:1333–1340. 15367555 10.1001/jama.292.11.1333

[2] Simmerman JM , Lertiendumrong J , Dowell SF , et al. The cost of influenza in Thailand. Vaccine. 2006;24:4417–4426. 16621187 10.1016/j.vaccine.2005.12.060

[3] Heikkinen T , Silvennoinen H , Peltola V , et al. Burden of influenza in children in the community. J Infect Dis. 2004;190:1369–1373. 15378427 10.1086/424527

[4] Cox NJ , Subbarao K . Influenza. Lancet. 1999;354:1277–1282. 10520648 10.1016/S0140-6736(99)01241-6

[5] Allison MA , Daley MF , Crane LA , et al. Influenza vaccine effectiveness in healthy 6‐ to 21‐month‐old children during the 2003–2004 season. J Pediatr. 2006;149:755–762. 17137887 10.1016/j.jpeds.2006.06.036

[6] Hurwitz ES , Haber M , Chang A , et al. Effectiveness of influenza vaccination of day care children in reducing influenza‐related morbidity among household contacts. JAMA. 2000;284:1677–1682. 11015798 10.1001/jama.284.13.1677

[7] Jefferson T , Di Pietrantonj C , Rivetti A , Bawazeer GA , Al‐Ansary LA , Ferroni E . Vaccines for preventing influenza in healthy adults. Cochrane Database Syst Rev. 2014;3:CD001269. 10.1002/14651858.CD001269.pub5 24623315

[8] Centers for Disease C, Prevention . Prevention and control of seasonal influenza with vaccines. Recommendations of the Advisory Committee on immunization practices–United States, 2013–2014. MMWR Recomm Rep. 2013;62:1–43. 24048214

[9] Feng L , Mounts AW , Feng Y , et al. Seasonal influenza vaccine supply and target vaccinated population in China, 2004–2009. Vaccine. 2010;28:6778–6782. 20688038 10.1016/j.vaccine.2010.07.064

[10] Yu H , Huang J , Huai Y , et al. The substantial hospitalization burden of influenza in central China: surveillance for severe, acute respiratory infection, and influenza viruses, 2010–2012. Influenza Other Respir Viruses. 2014;8:53–65. 24209711 10.1111/irv.12205 PMC4177798

[11] Li H , Ji CY , Zong XN , Zhang YQ . Body mass index growth curves Chinese children and adolescents aged 0 to 18 years. Chin J Pediatr. 2009;47:493–498. 19951508

[12] Nair H , Brooks WA , Katz M , et al. Global burden of respiratory infections due to seasonal influenza in young children: a systematic review and meta‐analysis. Lancet. 2011;378:1917–1930. 22078723 10.1016/S0140-6736(11)61051-9

[13] Kenmoe S , Tchendjou P , Vernet MA , et al. Viral etiology of severe acute respiratory infections in hospitalized children in Cameroon, 2011–2013. Influenza Other Respir Viruses. 2016;10:386–393. 27012372 10.1111/irv.12391 PMC4947949

[14] WHO . Vaccines against influenza. WHO position paper November 2012. 2012 Available at: http://www.who.int/immunization/position_papers/PP_influenza_november2012_summary.pdf. Accessed August 23, 2016.

[15] Usonis V , Anca I , André F , et al. Central European vaccination advisory group (CEVAG) guidance statement on recommendations for influenza vaccination in children. BMC Infect Dis. 2010;14:168. 10.1186/1471-2334-10-168 PMC2905419 20546586

[16] Fiore AE , Uyeki TM , Broder K , et al. Prevention and control of influenza with vaccines: recommendations of the advisory committee on immunization practices (ACIP), 2010. MMWR Recomm Rep. 2010;14:1–62. 20689501

[17] Joint Committee on Vaccination and Immunisation . JCVI statement on the annual influenza vaccination programme – extension of the programme to children. Available at: https://www.gov.uk/government/uploads/system/uploads/attachment_data/file/224775/JCVI-statement-on-the-annual-influenza-vaccination-programme-25-July-2012.pdf. Accessed August 23, 2016.

[18] CDC . Summary recommendations: prevention and control of influenza with vaccines: recommendations of the Advisory Committee on Immunization Practices (ACIP)—United States, 2013–14. Atlanta, GA: US Department of Health and Human Services, CDC; 2013 Available at: http://www.cdc.gov/flu/professionals/acip/2013-summary-recommendations.htm. Accessed July 1, 2015.

[19] Gilca R , Deceuninck G , De Serres G , et al. Effectiveness of pandemic H1N1 vaccine against influenza‐related hospitalization in children. Pediatrics. 2011;128:e1084–e1091. 21987710 10.1542/peds.2010-3492

[20] Yang Z , Dong Z , Fu C . Seasonal influenza vaccine effectiveness among children aged 6 to 59 months in southern China. PLoS One. 2012;7:e30424. 22291953 10.1371/journal.pone.0030424 PMC3265496

[21] He Q , Xu J , Chen X , et al. Effectiveness of seasonal influenza vaccine against clinically diagnosed influenza over 2 consecutive seasons in children in Guangzhou, China: a matched case‐control study. Hum Vaccin Immunother. 2013;9:1720–1724. 23733038 10.4161/hv.24980 PMC3906272

[22] Cowling BJ , Chan KH , Feng S , et al. The effectiveness of influenza vaccination in preventing hospitalizations in children in Hong Kong, 2009–2013. Vaccine. 2014;32:5278–5284. 25092636 10.1016/j.vaccine.2014.07.084 PMC4165553

[23] Ferdinands JM , Olsho LE , Agan AA , et al. Effectiveness of influenza vaccine against life‐threatening RT‐PCR‐confirmed influenza illness in US children, 2010–2012. J Infect Dis. 2014;210:674–683. 24676207 10.1093/infdis/jiu185

[24] Heinonen S , Silvennoinen H , Lehtinen P , et al. Early oseltamivir treatment of influenza in children 1–3 years of age: a randomized controlled trial. Clin Infect Dis. 2010;51:887–894. 20815736 10.1086/656408

[25] Han K , Ma H , An X , et al. Early use of glucocorticoids was a risk factor for critical disease and death from pH1N1 infection. Clin Infect Dis. 2011;53:326–333. 21810744 10.1093/cid/cir398

[26] Diaz E , Martin‐Loeches I , Canadell L , et al. Corticosteroid therapy in patients with primary viral pneumonia due to pandemic (H1N1) 2009 influenza. J Infect. 2012;64:311–318. 22240033 10.1016/j.jinf.2011.12.010

[27] Boudreault AA , Xie H , Leisenring W , et al. Impact of corticosteroid treatment and antiviral therapy on clinical outcomes in hematopoietic cell transplant patients infected with influenza virus. Biol Blood Marrow Transplant. 2011;17:979–986. 20870025 10.1016/j.bbmt.2010.09.014 PMC3676866

[28] WHO . Clinical management of human infection with pandemic (H1N1) 2009: revised guidance. Available at: http://www.who.int/csr/resources/publications/swineflu/clinical_management_h1n1.pdf?ua=1. Accessed July 1, 2015.

[29] Spellberg B , Guidos R , Gilbert D , et al. The epidemic of antibiotic‐resistant infections: a call to action for the medical community from the infectious diseases society of America. Clin Infect Dis. 2008; 46:155–164. 18171244 10.1086/524891

[30] Yu H , Yang W , Varma JK . To save children's lives, China should adopt an initiative to speed introduction of pneumonia. Health Aff. 2012;31:2545–2553. 10.1377/hlthaff.2011.1272 23129686

[31] DiFranza JR , Aligne CA , Weitzman M . Prenatal and postnatal environmental tobacco smoke exposure and children's health. Pediatrics. 2004;113(4 Suppl):1007–1015. 15060193

[32] Peterson EL , Johnson CC , Ownby DR . Use of urinary cotinine and questionnaires in the evaluation of infant exposure to tobacco smoke in epidemiologic studies. J Clin Epidemiol. 1997;50:917–923. 9291877 10.1016/s0895-4356(97)00095-4

[33] Okah FA , Choi WS , Okuyemi KS , Ahluwalia JS . Effect of children on home smoking restriction by inner‐city smokers. Pediatrics. 2002;109:244–249. 11826202 10.1542/peds.109.2.244

[34] WHO . Tobacco in China. Available at: http://www.wpro.who.int/china/mediacentre/factsheets/tobacco/en/. Accessed July 1, 2015.

