# Supplementary material for: Clinical characteristics and factors associated with severe acute respiratory infection and influenza among children in Jingzhou, China
Source: Influenza Other Respir Viruses. 2016 Sep 20;11(2):148–56. doi: 10.1111/irv.12419 (PMC5304575; doi:10.1111/irv.12419)
Supplement: Supplementary file 1 [file IRV-11-148-s001.docx]

Supplementary Table 1-1: Univariate analyses of risk factors associated with radiographically diagnosed pneumonia among pediatric SARI patients with lab-confirmed influenza aged 0-23 months, Jingzhou China, April 5, 2010 – April 8, 2012

| Characteristics^1^ | Case-patients with pneumonia (n=110) | Case-patients without pneumonia (n=261) | *p-value* |
| --- | --- | --- | --- |
| Male sex | 71 (65) | 153(59) | 0.287 |
| Median (IQR) age (months) | 9.6 (6-16.8) | 12 (6-16.8) | 0.135 |
| Age group |  |  |  |
| <6 months | 29 (26) | 50 (19) | 0.121 |
| >6 months | 81 (74) | 211 (81) |  |
| At least one underlying medical condition | 3 (3) | 6 (2) | 0.728 |
| Asthma | 0 | 0 | -- |
| Prematurity^2^ | 2 (2) | 4 (2) | 1.000 |
| Low birth weight^3^ | 1 (0.9) | 5 (2) | 0.674 |
| Vaccination history^4^ |  |  |  |
| Received monovalent pandemic H1N1 vaccination^5^ | 0 | 0 | --- |
| Received seasonal trivalent influenza vaccination^6^ | 10/48 (21) | 15/110 (14) | 0.254 |
| Exposure history |  |  |  |
| At least one tobacco smoker in household | 17/71 (24) | 43/140 (31) | 0.303 |
| Influenza virus type/subtype |  |  |  |
| Influenza B | Ref | ref | 0.606 |
| A(H1N1)pdm09 | 27 (25) | 46 (18) |  |
| A (H3N2) | 41 (37) | 131 (50) |  |
| *Antimicrobial use* |  |  |  |
| Antibiotics started before hospital admission | 41/77 (53) | 71/181 (39) | **<0.05** |
| Antibiotics given during hospitalization | 110 (100) | 259 (99) | 0.357 |
| Received corticosteroids during hospitalization | 16 (15) | 27 (10) | 0.248 |
| Received oseltamivir during hospitalization | 0 | 0 | - |

^1^Data presented as no. (%) of patients unless otherwise indicated. Denominators for testing of fewer patients than full group are indicated. Percentages may not total 100 because of rounding. IQR, interquartile range.

^2^Dfined as gestational age <37 weeks at birth for children aged <2 years.

^3^Defined as [birth weight](http://en.wikipedia.org/wiki/Birth_weight) of a live born infant of less than 2,500 g for children aged <2 years.

^4^Answered ‘Yes’ to this question, ‘During the past 12 months, have you had a flu shot?’ which were asked during a face-to-face interview.

^5^Of children aged ≥6 months eligible to receive seasonal trivalent inactive influenza vaccine.

^6^Of children aged ≥6 months eligible to receive monovalent pandemic A (H1N1) influenza vaccine.

Supplementary Table 1-2: Multivariable analyses of risk factors associated with presence of radiographic diagnosis of pneumonia among lab-confirmed pediatric SARI patients aged 0-23 months, Jingzhou China, April 5, 2010 – April 8, 2012

| Characteristic | OR (95% CI) | p-value^*^ |
| --- | --- | --- |
| Antibiotic use before hospital admission | 1.954 (1.123-3.401) | 0.018 |
| Influenza virus type/subtype | 0.997 (0.903-1.101) | 0.958 |

Supplementary Table 2-1: Univariate analyses of risk factors associated with radiographically diagnosed pneumonia associated with influenza among pediatric SARI patients aged 2-4 years old, Jingzhou, China, April 5, 2010 – April 8, 2012

| Characteristics of influenza patients^1^ | Case-patients with pneumonia (n=105) | Case-patients without pneumonia (n=195) | *p-value* |
| --- | --- | --- | --- |
| Male sex | 60 (57) | 115 (59) | 0.759 |
| Median (IQR) age (year) | 3.0 (2.5-3.7) | 3.0 (2.4-3.6) | 0.272 |
| At least one underlying medical condition | 3 (3) | 0 | **<0.05** |
| Obesity^2^ | 0 | 1 (0.5) | 0.876 |
| Duration from date of illness onset to hospital admission | 2 (0-3) | 3 (1-5) | **0.001** |
| Duration from hospital admission to discharge or death during hospitalization | 6 (5-8) | 5 (4-6) | **< 0.001** |
| Influenza virus type/subtype |  |  |  |
| Influenza B | ref | ref |  |
| A(H1N1)pdm09 | 14 (13) | 33 (17) | 0.339 |
| A (H3N2) | 47 (45) | 90 (46) |  |
| *Exposure history* |  |  |  |
| At least one tobacco smoker in household | 16/67 (24) | 42/131 (32) | 0.231 |
| Antimicrobial use |  |  |  |
| Antibiotics started before hospital admission | 54/77 (70) | 86/149 (58) | 0.069 |
| Antibiotics given during hospitalization | 105 (100) | 194 (100) | 0.462 |
| Received corticosteroids during hospitalization | 14 (13) | 17 (9) | 0.210 |
| Received oseltamivir during hospitalization | 1 (1) | 1 (0.5) | 0.655 |

^1^Data presented as no. (%) of patients unless otherwise indicated. Denominators for testing of fewer patients than full group are indicated. Percentages may not total 100 because of rounding. IQR, interquartile range.

^2^Body-mass index (BMI) was calculated for patients with available height and weight data to assess obesity using both Chinese criteria (BMI greater than the cut-off values for children aged 2-17 years). BMI was not calculated in children aged <2 years.

Supplementary Table 2-2: Multivariable analyses of risk factors associated with radiographically-diagnosed pneumonia among pediatric SARI patients with lab-confirmed influenza aged 2-4 years, Jingzhou China, April 5, 2010 – April 8, 2012

| Characteristic | OR (95% CI) | *p-value*^*^ |
| --- | --- | --- |
| Duration from date of illness onset to hospital admission | 1.176 (1.031-1.343) | 0.016 |
| Antibiotic use before hospital admission | 0.688 (0.373-1.271) | 0.233 |

Supplementary Table 3-1: Univariate analyses of risk factors associated with radiographically diagnosed pneumonia associated with influenza among pediatric SARI patients aged 5-15 years old, Jingzhou China, April 5, 2010 – April 8, 2012

| Characteristics of influenza patients^1^ | Case-patients with pneumonia (n=31) | Case-patients without pneumonia (n=105) | *p-value* |
| --- | --- | --- | --- |
| Male sex | 14 (45) | 64 (61) | 0.219 |
| Median (IQR) age (year) | 7.6 (5.7-9.0) | 6.7 (5.7-9.2) | 0.971 |
| At least one underlying medical condition | 1 (3) | 3 (3) | 1.000 |
| Obesity^2^ | 0 | 1 (1) | 0.694 |
| Median (IQR) days from date of illness onset to hospital admission | 3.0 (1.0-5.0) | 2.0 (1.0-4.0) | 0.270 |
| Influenza virus type/subtype |  |  |  |
| Influenza B | ref | ref |  |
| A(H1N1)pdm09 | 5 (16) | 9 (9) | **<0.05** |
| A (H3N2) | 11 (36) | 15 (14) |  |
| *Exposure history* |  |  |  |
| At least one tobacco smoker in household | 4/22 (18) | 17/51 (33) | 0.189 |
| *Antimicrobial use* |  |  |  |
| Antibiotics started before hospital admission | 17/23 (74) | 60/81 (74) | 0.988 |
| Antibiotics given during hospitalization | 30 (97) | 105 (100) | 0.228 |
| Received corticosteroids during hospitalization | 5 (16) | 10 (10) | 0.302 |
| Received oseltamivir during hospitalization | 0 | 0 | - |

^1^Data is presented as no. (%) of patients unless otherwise indicated. Denominators for testing of fewer patients than full group are indicated. Percentages may not total 100 because of rounding. IQR, interquartile range.

^2^Body-mass index (BMI) was calculated for patients with available height and weight data to assess obesity using both Chinese criteria (BMI greater than the cut-off values for children aged 2-17 years). BMI was not calculated in children aged <2 years.

Supplementary Table 3-2: Multivariable analyses of risk factors associated with radiographic diagnosis of pneumonia among pediatric SARI patients aged 5-15 years with lab-confirmed influenza, Jingzhou China, April 5, 2010 – April 8, 2012

| Characteristic | OR (95% CI) | *p-value*^*^ |
| --- | --- | --- |
| Influenza A virus subtype |  |  |
| A (H3N2) | 1.758 (0.325-9.591) | 0.513 |
| A(H1N1)pdm09 | 2.636 (0.456-15.248) | 0.279 |
| Antibiotics started before hospital admission | 0.720 (0.177-2.930) | 0.646 |
| At least one tobacco smoker in household | 4.308 (0.864-21.489) | 0.075 |

Supplementary Table 4: Univariate analyses of risk factors associated with influenza among pediatric SARI patients aged <6 months, Jingzhou, China, April 5, 2010 – April 8, 2012

| Characteristics^1^ | SARI patients with influenza  (n=156) | SARI patients without influenza  (n=1,099) | *p*-value |
| --- | --- | --- | --- |
| Male sex | 96 (62) | 655 (60) | 0.644 |
| Median (IQR) age (months) | 4.8 (2.4-4.8) | 4.8 (2.4-4.8) | 0.919 |
| Prematurity^2^ | 3 (2) | 16 (2) | 0.655 |
| Low birth weight^3^ | 3 (2) | 5 (0.5) | 0.066 |
| At least one tobacco smoker in household | 31/109 (28) | 178/804 (22) | 0.142 |
| Contact with anyone else with fever or respiratory symptoms^4^ | 7/110 (6) | 46/732 (6) | 0.974 |
| More than one child living in household in past 3 months | 10 (6) | 46 (4) | 0.208 |

^1^Data presented as no. (%) of patients unless otherwise indicated. Denominators for testing of fewer patients than full group are indicated. Percentages may not total 100 because of rounding. IQR, interquartile range.

^2^Dfined as gestational age <37 weeks at birth for children aged <2 years.

^3^Defined as [birth weight](http://en.wikipedia.org/wiki/Birth_weight) of a live born infant of less than 2,500 g for children aged <2 years.

^4^Contact with anyone else with fever or respiratory symptoms defined as having been in close contact (within one meter) or direct contact of person with fever or respiratory symptoms in the previous 2 weeks.

Supplementary Table 5: Univariate analyses of risk factors associated with influenza among pediatric SARI patients aged 6-23 months, Jingzhou, China, April 5, 2010 – April 8, 2012

| Characteristics^1^ | SARI patients with influenza  (n=602) | SARI patients without influenza virus infection  (n=5,726) | *p-value* |
| --- | --- | --- | --- |
| Male sex | 354 (59) | 3,355 (59) | 0.920 |
| Median (IQR) age (months) | 12 (9.6-18) | 12 (9.6-16.8) | 0.188 |
| At least one underlying medical condition | 20 (3) | 131 (2) | 0.114 |
| Asthma | 1 (0.2) | 9 (0.2) | 0.958 |
| Prematurity^2^ | 14 (2) | 81 (1) | 0.080 |
| Low birth weight^3^ | 10 (2) | 62 (1) | 0.203 |
| *Received Influenza vaccination^4^* | | | |
| Received monovalent pandemic H1N1 vaccination^5^ | 0 | 18/1,047 (0.2) | 0.093 |
| Received seasonal trivalent influenza vaccination^6^ | 48/368 (13) | 495/3,589 (14) | 0.691 |
| *Exposure history* |  |  |  |
| At least one tobacco smoker in household | 118/410 (29) | 901/4,103 (22) | **<0.05** |
| Sick contact with fever or respiratory symptoms^7^ | 34/387 (9) | 208/3,814 (6) | **<0.05** |
| More than one child living in household in past 3 months | 19 (3) | 163 (3) | 0.666 |

^1^Data presented as no. (%) of patients unless otherwise indicated. Denominators for testing of fewer patients than full group are indicated. Percentages may not total 100 because of rounding. IQR, interquartile range.

^2^Dfined as gestational age <37 weeks at birth for children aged <2 years.

^3^Defined as [birth weight](http://en.wikipedia.org/wiki/Birth_weight) of a live born infant of less than 2,500 g for children aged <2 years.

^4^Answered ‘Yes’ to this question, ‘During the past 12 months, have you had a flu shot?’ which were asked during a face-to-face interview.

^5^Of children aged ≥6 months eligible to receive seasonal trivalent inactive influenza vaccine.

^6^Of children aged ≥6 months eligible to receive monovalent pandemic A (H1N1) influenza vaccine.

^7^Contact with anyone else with fever or respiratory symptoms defined as having been in close contact (within one meter) or direct contact of person with fever or respiratory symptoms in the previous 2 weeks.

Supplementary Table 6: Univariate analyses of risk factors associated with influenza among pediatric SARI patients aged 2-4 years, Jingzhou, China, April 5, 2010 – April 8, 2012

| Characteristics^1^ | SARI patients with influenza  (n=669) | SARI patients without influenza  (n=4,361) | *p-value* |
| --- | --- | --- | --- |
| Male sex | 381 (57) | 2,359 (54) | 0.167 |
| Median (IQR) age (years) | 3.0 (2.5-3.9) | 2.9 (2.5-3.6) | **<0.01** |
| At least one underlying medical condition | 6 (0.9) | 102 (2) | **<0.05** |
| Asthma | 1 (0.1) | 18 (0.4) | 0.301 |
| Chronic bronchitis | 0 | 5 (0.1) | 1.000 |
| Obesity^2^ | 2 (0.3) | 8 (0.2) | 0.902 |
| *Received Influenza vaccination^3^* | | | |
| Received monovalent pandemic H1N1 vaccination^4^ | 2/171 (0.1) | 29/929 (3) | 0.210 |
| Received seasonal trivalent influenza vaccination^5^ | 76/410 (19) | 471/2,698 (18) | 0.593 |
| *Exposure history* |  |  |  |
| At least one tobacco smoker in household | 105/455 (23) | 628/3,061 (21) | 0.210 |
| Sick contact with fever or respiratory symptoms^6^ | 28/407 (7) | 99/2,709 (4) | **<0.05** |
| More than one child living in household in past 3 months | 23 (3) | 112 (3) | 0.195 |

^1^Data presented as no. (%) of patients unless otherwise indicated. Denominators for testing of fewer patients than full group are indicated. Percentages may not total 100 because of rounding. IQR, interquartile range.

^2^Body-mass index (BMI) was calculated for patients with available height and weight data to assess obesity using Chinese criteria (BMI greater than the cut-off values for children aged 2-17 years). BMI was not calculated in children aged <2 years.

^3^Answered ‘Yes’ to this question, ‘During the past 12 months, have you had a flu shot?’ which were asked during a face-to-face interview.

^4^Of children aged ≥6 months eligible to receive seasonal trivalent inactive influenza vaccine.

^5^Of children aged ≥6 months eligible to receive monovalent pandemic A (H1N1) influenza vaccine.

^6^Contact with anyone else with fever or respiratory symptoms defined as having been in close contact (within one meter) or direct contact of person with fever or respiratory symptoms in the previous 2 weeks.

Supplementary Table 7: Univariate analyses of risk factors associated with influenza among pediatric SARI patients aged 5-15 years, Jingzhou, China, April 5, 2010 – April 8, 2012

| Characteristics^1^ | SARI patients with influenza  (n=347) | SARI patients without influenza  (n=1,519) | | *p-value* |
| --- | --- | --- | --- | --- |
| Male | 193 (56) | 879 (58) | | 0.445 |
| Median (IQR) age (years) | 6.7 (5.8-8.5) | 6.9 (5.8-8.8) | | 0.506 |
| At least one underlying medical condition | 9 (3) | 27 (2) | | 0.319 |
| Asthma | 1 (0.3) | 7 (0.5) | | 1.000 |
| Chronic bronchitis | 0 | 1 (0.1) | | 1.000 |
| Obesity^2^ | 2 (0.6) | 8 (0.5) | | 0.931 |
| *Received Influenza vaccination^3^* | | | | |
| Received monovalent pandemic H1N1 vaccination^4^ | 3/59 (5) | 29/314 (9) | 0.446 | |
| Received seasonal trivalent influenza vaccination^5^ | 27/181 (15) | 150/867 (17) | 0.436 | |
| *Exposure history* |  |  | |  |
| At least one tobacco smoker in household | 54/208 (26) | 158/960 (16) | | **<0.05** |
| Sick contact with fever or respiratory symptoms^6^ | 13/179 (7) | 37/823 (4) | | 0.123 |
| More than one child living in household in past 3 months | 8 (2) | 23 (2) | | 0.348 |

^1^Data presented as no. (%) of patients unless otherwise indicated. Denominators for testing of fewer patients than full group are indicated. Percentages may not total 100 because of rounding. IQR, interquartile range.

^2^Body-mass index (BMI) was calculated for patients with available height and weight data to assess obesity using Chinese criteria (BMI greater than the cut-off values for children aged 2-17 years). BMI was not calculated in children aged <2 years.

^3^Answered ‘Yes’ to this question, ‘During the past 12 months, have you had a flu shot?’ which were asked during a face-to-face interview.

^4^Of children aged ≥6 months eligible to receive seasonal trivalent inactive influenza vaccine.

^5^Of children aged ≥6 months eligible to receive monovalent pandemic A (H1N1) influenza vaccine.

^6^Contact with anyone else with fever or respiratory symptoms defined as having been in close contact (within one meter) or direct contact of person with fever or respiratory symptoms in the previous 2 weeks.

Supplementary Table 8. Multivariable analyses of factors significantly associated with influenza among pediatric SARI patients, Jingzhou, China, April 5, 2010 – April 8, 2012

| Characteristic | OR (95% CI) | *p-value*^*^ |
| --- | --- | --- |
| 0-6 months |  |  |
| Low birth weight^1^ | 6.081 (1.308-28.266) | <0.05 |
| 6-23 months |  |  |
| Anyone in household smoke cigarettes | 1.363 (1.047-1.776) | <0.05 |
| 2-4 years |  |  |
| Median (IQR) age (years) | 1.314 (1.144-1.509) | <0.001 |

*Multivariable logistic regression analyses were performed. Variable selection based on the significant p-value less than 0.1 from univariate analyses.

^1^Defined as [birth weight](http://en.wikipedia.org/wiki/Birth_weight) of a live born infant of less than 2,500 g for children aged <2 years.
